# Reconstitution of native intrinsic conduction in patients with chronic conduction block with His bundle pacing

**DOI:** 10.1016/j.hrcr.2021.03.006

**Published:** 2021-03-23

**Authors:** Fatima M. Ezzeddine, Rajeev Singh, Samuel J. Asirvatham, Gopi Dandamudi, Subodh R. Devabhaktuni

**Affiliations:** ∗ Mayo Clinic, Rochester, Minnesota; † Piedmont Health, Atlanta, Georgia; ‡ CHI Franciscan, Tacoma, Washington; § University of Arkansas Medical Sciences, Little Rock, Arkansas

**Keywords:** Bundle branch block, Electrocardiography, Electrophysiology, His bundle pacing, Native conduction

## Introduction

His bundle pacing (HBP) has emerged as a physiological form of ventricular pacing that has been shown to be safe and feasible in clinical practice. Since it induces ventricular contraction by exciting the intrinsic conduction system, it has the benefit of reducing or eliminating both interventricular and intraventricular dyssynchrony. In patients with infranodal block^1^ or left bundle branch block (LBBB),^2^ HBP can result in normalization of QRS complex, ventricular activation, and hemodynamic parameters including left ventricular (LV) systolic function. We present 2 cases where HBP not only normalized ventricular activation but also reconstituted native intrinsic conduction after years of dormancy.

## Case report

### Case 1

A 76-year-old woman was diagnosed with complete infra-Hisian heart block by an electrophysiology study and underwent dual-chamber pacemaker implantation. Three years later, she presented with sudden cardiac arrest (in the setting of preserved LV systolic function) that was attributed to possible long QT syndrome. At that time, her pulse generator was upgraded to a dual-chamber implantable cardioverter-defibrillator (ICD). Nine years later, the patient developed heart failure symptoms including dyspnea on exertion and lower extremity edema. A year prior, she had been diagnosed with breast cancer and completed a course of doxorubicin chemotherapy. Her echocardiogram demonstrated a LV ejection fraction (LVEF) of 35%. She was placed on guideline-directed medical therapy for heart failure including beta blockers, angiotensin-converting enzyme inhibitors, and mineralocorticoid receptor antagonists. She continued to have reduced LV systolic function but deferred to undergo upgrade to cardiac resynchronization therapy (CRT) owing to chronic right ventricular (RV) pacing.

**Figure 1:**
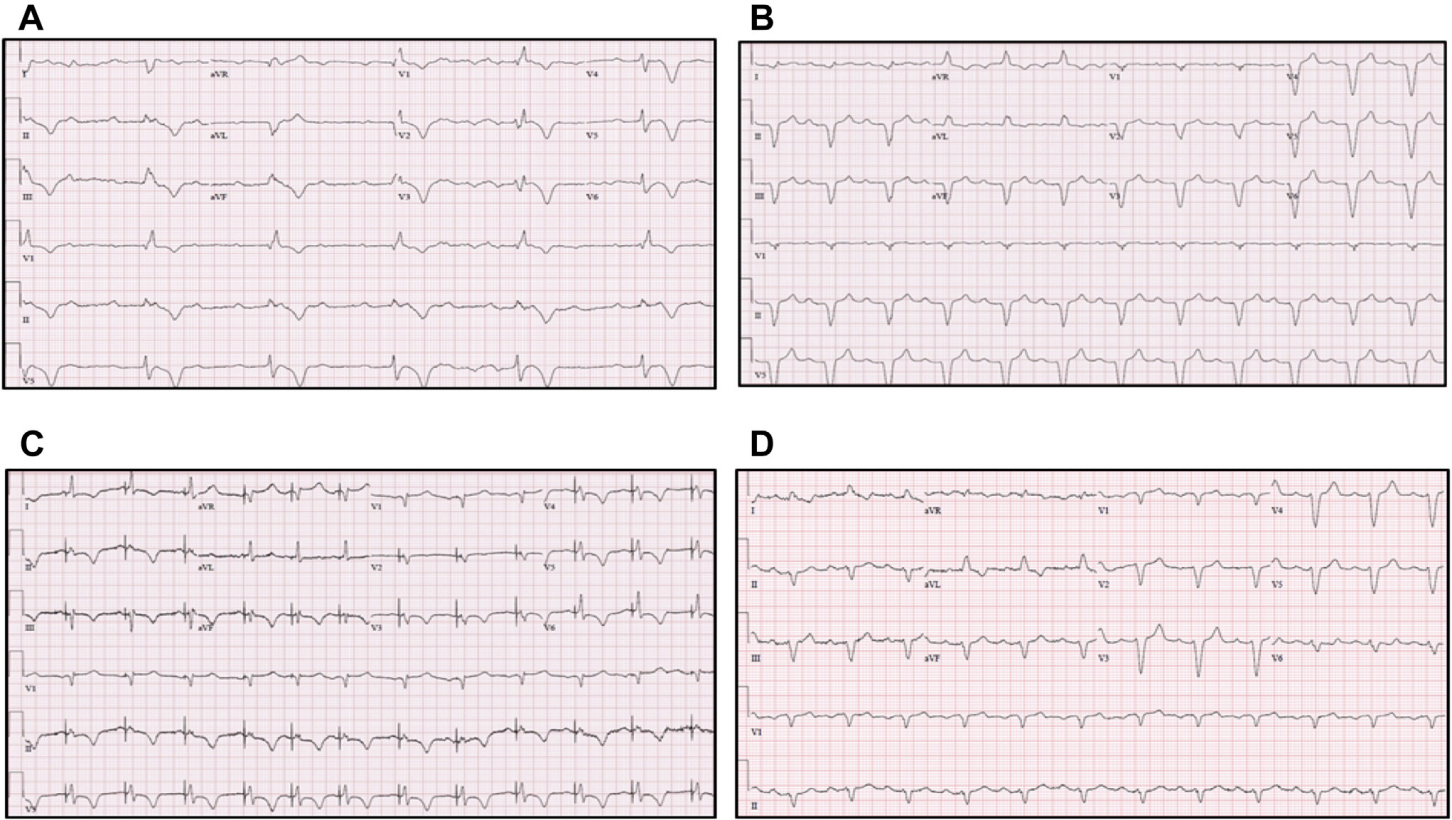
**A:** Electrocardiogram demonstrating complete heart block with a right bundle branch block escape pattern. **B:** Ventricular paced electrocardiogram obtained prior to lead fracture. Note the wide left bundle branch block pattern. **C:** Electrocardiogram demonstrating non-selective His bundle pacing immediately after the procedure was completed. Note the T-wave inversions due to T-wave memory phenomenon. The T-wave inversions normalized 4 weeks after the procedure. **D:** Electrocardiogram demonstrating restoration of AV conduction when pacing was turned off.

Three years later, she presented to the Emergency Department with lightheadedness on activity. Her electrocardiogram demonstrated bradycardia with complete AV block with a right bundle branch block pattern escape rhythm (Figure 1A). Further workup revealed that the patient’s RV lead had fractured, resulting in failure to capture. She had a slow escape rhythm requiring emergent transvenous ventricular pacing overnight. On telemetry, she had no intrinsic AV conduction prior to RV lead revision and her prior device checks had shown no intrinsic AV conduction.

Given the presence of complete infra-Hisian AV block on prior study, heart failure with reduced ejection faction in the setting of wide left bundle branch RV pacing morphology (Figure 1B), it was decided to upgrade her pulse generator to a biventricular ICD via HBP. His-bundle mapping was performed using a C315 His sheath and a 3830 pacing lead (Medtronic, Minneapolis, MN).^3^ Non-selective HBP was achieved at an output of 1 V @ 1 ms (Figure 1C). The His bundle lead was connected to the LV port and the RV single-coil ICD lead was connected to the RV port. LV-to-RV offset was programmed to 80 ms to promote HBP while minimizing the possible effects of fusion. The patient reported significant improvement in her symptoms (from NYHA class II to class I), and a repeat cardiac echocardiogram 3 months later demonstrated normalization of LV systolic function (echo prior to RV lead revision had demonstrated an LVEF of 35%). Owing to the rapid reversal of LV systolic dysfunction, it was felt that her cardiomyopathy was primarily related to pacing-induced cardiomyopathy. Several months later, she remained symptom-free, with no functional limitations. During routine device check, RV pacing inhibition demonstrated 1:1 AV conduction with the presence of a LBBB (Figure 1D). She maintained 1:1 AV conduction with LBBB during her subsequent device checks over the next 2 years.

### Case 2

**Figure 2:**
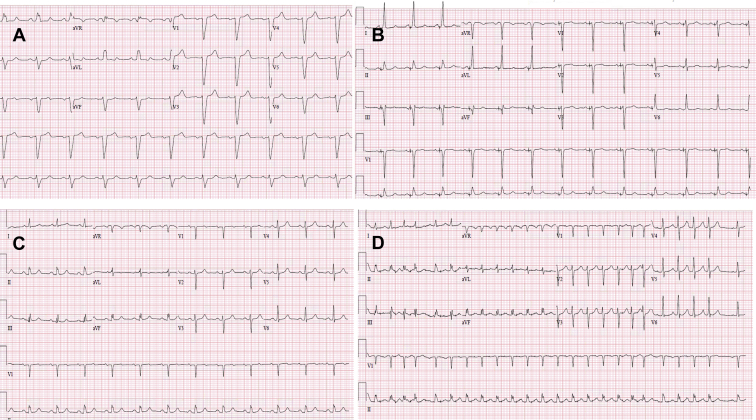
**A:** Electrocardiogram demonstrating left bundle branch block (LBBB) prior to His bundle pacing. **B:** Ventricular-paced electrocardiogram obtained post His bundle pacing. Note the resolution of the LBBB. **C:** Electrocardiogram showing restoration of the LBBB conduction when pacing was turned off. **D:** Burst atrial pacing with no evidence of rate-related LBBB.

A 51-year-old woman with a past medical history of hypertension and diabetes mellitus presented with shortness of breath and leg swelling. She was diagnosed with idiopathic non-ischemic cardiomyopathy at that time after undergoing cardiac catheterization and genetic testing. Her LVEF progressively deteriorated over subsequent years to 20%. She was also noted to have wide LBBB (>150 ms) (Figure 2A). The patient’s NYHA class II heart failure symptoms never improved despite being managed with optimal guideline-directed medical therapy for several months, which led to the decision of pursuing a CRT defibrillator in the setting of a LVEF <30% and wide LBBB. She had declined a CRT defibrillator for 3 years prior to agreeing to undergo device implantation.

After discussing her options, she agreed to undergo HBP with the hope of recruiting her underlying LBBB. During the procedure, a C315 His sheath was advanced to the tricuspid annulus and His bundle mapping was performed. This demonstrated excellent His bundle recordings with normal AH and HV intervals. The pacing lead was screwed into place with excellent selective HBP and complete recruitment of the intrinsic LBBB at 0.6 V at 1.0 ms (Figure 2B). On follow-up, the patient reported significant improvement in her symptoms (from NYHA class II to class I), and a repeat cardiac echocardiogram 3 months later demonstrated normalization of her LV systolic function. Pacing thresholds remained stable after 3 months at 0.5 V at 1 ms. During her 1-month follow-up post implantation, pacing inhibition demonstrated an intrinsic narrow QRS complex with 1:1 AV conduction (Figure 2C). In addition, burst atrial pacing and atrial extrastimulus testing through the device did not induce rate-related LBBB (Figure 2D).

## Discussion

Cardiac stimulation is initiated by the autonomous electric discharges of the sinoatrial node. This automatic pacemaker activity also occurs, though at a slower rate, in other subsidiary pacemaker cells located in the atrioventricular node, the His bundle, and the Purkinje fibers. The mechanisms involved in cardiac conduction initiation and propagation have been the subject of investigation for decades. Extensive analysis of the cardiac pacemaker activity has shown that cardiac pacemaker cells are sensitive to discrete stimuli, and this sensitivity depends on the timing, amplitude, and duration of the stimulus.^4^ Consequently, repetitive stimuli can force the pacemaker to discharge at rates that may be slower or faster than its own rate. As such, and in the case of a proximal defect in the conduction system, electrical stimulation of these subsidiary pacemaker cells can depolarize them and generate an action potential that propagates along an intact distal conduction system, restoring the inherent cardiac conduction. This led to the emergence of HBP as an attractive true physiological form of pacing that utilizes the native conduction system.

### Propagation of the action potential: Source-sink mechanism

When a depolarizing current pulse is applied during pacing, a voltage gradient is created between the cell that has fired the action potential and the other cells downstream. The inward depolarizing current associated with the action potential upstroke acts as a source of electrical charge to the cells downstream that act as a current sink. Therefore, the depolarizing current should be large enough to bring the membrane above the threshold for sodium current activation and response occurs when the source and the sink have matching electrophysiological properties that underlie excitability, refractoriness, and cell-to-cell communication.

Of note, a repolarizing outward current at the distal cells always opposes the depolarizing force of the current source. This is illustrated by the concept of liminal length that was described in 1937 by Rushton.^4^ Therefore, success or failure of propagation also depends on whether the density of the depolarizing current can overcome the repolarizing current downstream. Interestingly, HBP has been shown to restore conduction not only in patients with conduction diseases that are involving the AV node but also in patients with infranodal block (case 1) and bundle branch block (case 2). This implies the presence of other mechanisms that come into play besides the source-sink mechanism.

### Longitudinal dissociation of the His bundle

In 1919, Kaufmann and Rothberger first proposed the concept of functional longitudinal dissociation of the His bundle.^5^ Based on this concept, Purkinje cells conduct in a longitudinal rather than transverse direction and predestined fibers within the His bundle conduct to each fascicle. This was demonstrated by James and Sherf,^6^ who studied the structure of the human His bundle under both light and electron microscopy, and found that twisting collagen strands in the AV node funnel into a more parallel orientation in the His bundle. Similarly, the Purkinje cells were longitudinally oriented with sparse transverse connections among them. In 1977, Narula^7^ showed that pacing distal to the site of conduction delay within the His bundle could recruit fibers predestined to the bundle branches, resulting in QRS narrowing.

Consequently, bundle branch block and complete infranodal block originating within the proximal His bundle can be bypassed by distal HBP, which supports this theory of longitudinal dissociation and pacing distal to a block, as illustrated in cases 1 and 2.

### Virtual electrode polarization

The virtual electrode polarization effect plays a key role in pacing and defibrillation. According to this concept, the delivery of charge to an area creates virtual electrodes with depolarized and hyperpolarized regions surrounding the pacing tip, which can lead to recovery of diseased local tissue from inexcitability. In this case, the pacing lead does not have to be located distal to a block to restore conduction, as it can induce recovery of a diseased tissue by delivering these virtual electrodes. This concept can also explain action potential propagation complementing the source-sink mechanism, which was previously described.

In some cases, HBP results in partial QRS narrowing without complete normalization, and this can be explained by distal or complex blocks within the His-Purkinje system, anatomical variations, lead placement, or a combination of these factors.

### Conducting beyond expectations

In addition to normalization of conduction block with HBP, our cases are, to our knowledge, the first report of patients who recovered areas of native conduction pathways after a period of continuous HBP. Case 1 illustrates return of AV conduction in a patient with longstanding infra-Hisian heart block (>8 years), while case 2 illustrates recovery of left bundle branch conduction in a patient with longstanding LBBB (>4 years). Both patients continue to have native intrinsic conduction several months post-procedure. This demonstrates new theoretical benefits of HBP: the ability to promote conduction down the native pathways that were previously non-conducting. Although the exact mechanism is not known, it is likely that some His-Purkinje conduction diseases are functional rather than pathologic. It is also possible that some His-Purkinje conduction diseases are paroxysmal rather than permanent. We hypothesize that the following mechanisms might have played a role in our cases. In case 1, this could be related to Prinzmetal facilitation, where subthreshold stimulus *distal* to a site or delay of block can facilitate antegrade conduction.^8^^,^^9^ In case 2, it could be related to Wedensky facilitation,^10^ where an impulse arriving at a blocked zone enhanced the excitability of the nerve beyond the block by lowering of threshold, thereby promoting antegrade conduction. An alternate mechanism is fibrosis from the pacing lead that increases the sink side impedance and thus optimizes the source and sink match.^11^ Another possibility could be that the HBP lead could have been screwed into the cells, which may have been causing electrotonic inhibition of the downstream cells. Another mechanism might be based on the liminal length theory: pacing stimuli at the His bundle at a higher strength could have overcome the opposing effect of repolarization.^4^ This hypothesis would not explain the persistent normal native conduction in the absence of HBP. However, persistent HBP could have led to upregulation of sodium ion channels in the prior non-conducting cells, resulting in restoration of conduction (electrical reverse remodeling). This might be similar to autonomic remodeling, leading to prolonged antiarrhythmic effect of epicardial injection of botulinum toxin on atrial fibrillation.^12^^,^^13^ Lastly, reversal of conduction abnormalities has been described after improvement in LV systolic function. There are a few case reports in the literature in which LBBB recovered to normal conduction after improvement in LV systolic function with medical therapy^14^ and/or biventricular pacing.^15^ Mechanical injury owing to increased wall stress and ventricular size was thought to be the cause of conduction disturbances in these cases. Some of these are theoretical concepts and need to be confirmed by animal model studies.

## Conclusion

In conclusion, HBP not only normalizes ventricular activation but can also restore native intrinsic conduction in some cases after several years of chronic conduction block. The exact frequency of this occurrence and underlying mechanisms are still not well known and need further investigation.

**Key Teaching Points**

• His bundle pacing can restore native intrinsic conduction in some cases after several years of chronic conduction block.
• Several mechanisms can explain recovery of native intrinsic conduction with His bundle pacing in patients with chronic conduction block, including Prinzmetal facilitation, Wedensky facilitation, source-sink match optimization due to fibrosis from the pacing lead, electrotonic inhibition of the downstream cells, and liminal length theory.
• The exact frequency of this occurrence and underlying mechanisms are still not well known and need further investigation.
